# Conversion Characteristics of Some Major Cannabinoids from Hemp (*Cannabis sativa* L.) Raw Materials by New Rapid Simultaneous Analysis Method

**DOI:** 10.3390/molecules26144113

**Published:** 2021-07-06

**Authors:** Byeong Ryeol Ryu, Md. Jahirul Islam, Md. Obyedul Kalam Azad, Eun-Ji Go, Md. Hafizur Rahman, Md. Soyel Rana, Young-Seok Lim, Jung-Dae Lim

**Affiliations:** 1 Department of Bio-Health Convergence, Kangwon National University, Chuncheon 24341, Korea; fbqudfuf0419@naver.com (B.R.R.); jahirulislam213@gmail.com (M.J.I.); azadokalam@gmail.com (M.O.K.A.); a01040363654@daum.net (E.-J.G.); hafizknu94@gmail.com (M.H.R.); soyelrana98@gmail.com (M.S.R.); 2 Physiology and Sugar Chemistry Division, Bangladesh Sugarcrop Research Institute, Ishurdi, Pabna 6620, Bangladesh

**Keywords:** cannabis, decarboxylation, cannabinoid, chromatography, cannabidiol

## Abstract

This study was carried out to develop a high-performance liquid chromatography method for short-time analysis of the main cannabinoids in the inflorescence of hemp (*Cannabis sativa* L.). We also performed decarboxylation of the raw material using our advanced analysis technique. In this study, the UV spectrum was considered to analyze each of the four common cannabinoids, solvents, and samples, where the uniform elution of acidic cannabinoids without peak tailing and acids was tested. Optimal results were obtained when readings were taken at a wavelength of 220 nm using water and methanol containing trifluoroacetic acid as mobile phases in a solvent gradient system. The established conditions were further validated by system suitability, linearity, precision, detection limit, and quantitation limit tests. The decarboxylation index (DT_50_) confirmed that Δ9-THCA decarboxylated faster than CBDA, and both maintained a linear relationship with time and temperature. In addition, the loss of cannabidiol was better prevented during the decarboxylation process in the natural state than in the extracted state.

## 1. Introduction

Linnaeus classified hemp in Europe as a single species (*Cannabis sativa* L.) [1], and de Lamarck separately classified Indian varieties as *Cannabis* *indica* Lam. [2]. Janischevsky classified Russian-born hemp as *Cannabis* *ruderalis* Jansich. without any characteristics [3]. Even today, it remains controversial whether all cannabis should be viewed as a single species of *C. sativa* or as multiple species divided into *C. sativa*, *C. indica*, and *C. ruderalis* [4]. For convenience, it is indicated as *‘Cannabis sativa* L.’.

**Figure 1:**
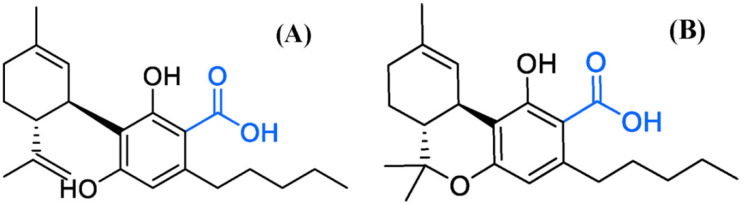
Carboxyl group of acid cannabinoids. (**A**) Cannabidiolic acid, (**B**) ∆9-tetrahydrocannabinolic acid. Carboxyl groups are removed by heat treatment and converted into neutral cannabinoids.

Hemp (*Cannabis sativa* L.) is an annual herb belonging to the Cannabaceae family, first recorded in China for the purpose of obtaining fiber [5]. Since then, it has been exploited for medical purposes for more than 10,000 years [6]. As many countries have approved hemp for medical applications, there has been a surge in the use of hemp drug therapy for pain relief [7]. Hemp contains more than 525 compounds and is known to express approximately 109 cannabinoids [8]. Cannabinoid refers to the C21 terpenophenol compound uniquely found in hemp [9]. Although expressed in small amounts in leaves, cannabinoid is mostly concentrated in the trichome part distributed in the bracts of the female flower of the plant. Its concentration is higher in the stalked-type trichome among the two types, ‘sessile’ and ‘stalked’ [10]. They are mostly present in the resin secreted from the trichomes of female plants, while male *C. sativa* have few glandular trichomes that can produce small amounts of psychoactive molecules [11]. The most abundant of these are the ∆9-tetrahydrocannabinol (∆9-THC) and cannabidiol (CBD) families [12]. The compound ∆9-THC is a major psychotropic cannabinoid [13] known to induce transient psychotic and anxiety symptoms [14,15]. It was recently found to be effective in treating Alzheimer’s disease and glaucoma [5]. CBD has an effect on anti-anxiety and anti-psychiatric diseases [16,17], and more than 1000 CBD-containing products are available in the market. It is thought to contain ingredients that can cure mental illness [18]. Each of these neutral cannabinoids is formed by non-enzymatic decarboxylation of acidic precursor cannabinoids called Δ9-tetrahydrocannabinolic acid (∆9-THCA) and cannabidiolic acid (CBDA) (Figure 1) [19,20], and heating temperature and time are known to significantly influence this decarboxylation process [21].

In this study, high-performance liquid chromatography (HPLC) was used to efficiently and accurately quantify four major cannabinoids (∆9-THC, ∆9-THCA, CBD, and CBDA) that can be naturally and artificially converted. These compounds exhibit physiological activities. Simultaneous analysis conditions were developed, established, and employed to study the decarboxylation of cannabis inflorescence.

## 2. Materials and Methods

### 2.1. Standard and Sample Preparation

A sample of the inflorescence part of cannabis (*Cannabis sativa* L.) was received from the Korea Cannabis Genetic Resource Center (Chuncheon, Korea). An inflorescence sample of a plant grown for 105 days (50 days after flowering treatment) in a nutrient solution cultivation system after sowing was used. The standard products cannabidiol (CBD) and Δ9-tetrahydrocannabinol (∆9-THC) were obtained from CAYMAN (Ann Arbor, MI, USA), the cannabidiolic acid (CBDA) standard product was from Cerilliant (Round Rock, TX, USA), and the Δ9-tetrahydrocannabinolic acid (∆9-THCA) standard product was manufactured by Sigma-Aldrich (St. Louis, MO, USA).

The inflorescence of freeze-dried hemp was pulverized using a chopper (YPT-1402, Young Polymer Tech Co., Incheon, Korea) certified by Korea Conformity Laboratories and filtered through a 12-mesh test sieve. The furnace particles were found to be homogeneous. In brief, 0.1 g of hemp sample was sonicated (JEIO TECH Co., Ltd., Daejeon, Korea) for 30 min with 30 mL extra pure grade MeOH (Daejung Chemicals and Metals Co., Ltd., Siheung, Korea) as an extraction solvent. After ultrasonic extraction at room temperature (23 ± 2 °C), the supernatant was sampled, filtered using a 0.45-micrometer syringe filter (Advantec, Tokyo, Japan), and used for HPLC analysis.

All solutions except CBDA in stock state were precisely weighed and then diluted using MeOH. The standard products and samples used in this study were received and tested with the approval of the Seoul Regional Food and Drug Administration (approval number: 1806).

#### 2.1.1. HPLC Condition Setting

HPLC was performed using a Shimadzu LC-20AT system with an SPD-20A detector (UV-Vis, Shimadzu Co., Kyoto, Japan) and a Zorbax SB-C18 column (Rapid Resolution, 4.6 × 100 mm, 3.5 μm, Agilent Technologies, Inc., Santa Clara, CA, USA). HPLC-grade ultrapure water, methanol (CH_3_OH), and acetonitrile (CH_3_CN) were purchased from Mallinckrodt Baker (Phillipsburg, NJ, USA). The ∆9-THC, ∆9-THCA, CBD, and CBDA standards were mixed at a final concentration of 100 μg/mL and subjected to analysis.

To determine the absorption region of each component, the absorbance spectrum was measured in the UV region of 190–400 nm. The spectra of methanol and acetonitrile were also measured in the same region to avoid the cut-off section of the solvent.

We analyzed the mixture of ultrapure water and selected organic solvents at concentrations of 60%, 70%, 80%, and 90% to improve kurtosis and skewness by adjusting pKa. Formic acid (FA, Merck, Darmstadt, Germany), trifluoroacetic acid (TFA, Sigma-Aldrich Co., St. Louis, MO, USA), and phosphoric acid (PA, Junsei Chemical Co., Ltd., Tokyo, Japan) were added at concentrations of 0.01%, 0.05%, and 0.10%, respectively, to explore optimal mobile conditions.

#### 2.1.2. Validation for Establishing Analysis Conditions

To determine the suitability of the developed analysis conditions, tests of the system suitability, specificity, linearity, range, accuracy, precision, detection limit, and quantitation limit were conducted with reference to the ICH guideline Q2 (R1) [22]. The United States Pharmacopeia (USP) was used to determine the suitability of the system.

### 2.2. Investigation of the Correlation between Hemp Inflorescence and Decarboxylation

Samples ground in the same manner as previously described were placed in a preheated, closed, dry oven (Sungchan Science Co., Pocheon, Korea) and sampled at 5-min intervals for 60 min. The temperature conditions were 90, 105, 120, and 135 °C.

To compare the decarboxylation of the extract and the natural product, two different types of cannabis extract were additionally prepared, extracted by applying the above extraction method, and concentrated at 30 °C using a rotary evaporator (Tokyo Rikakikai). The moisture was removed using a freeze-dryer (FD8512, IlShinBioBase Co., Ltd., Dongducheon, Korea). After decarboxylation at 135 °C for 30 min in the absence of any solvent, the product was recovered with MeOH and used for analysis.

## 3. Results

### 3.1. HPLC Condition Setting

**Figure 2:**
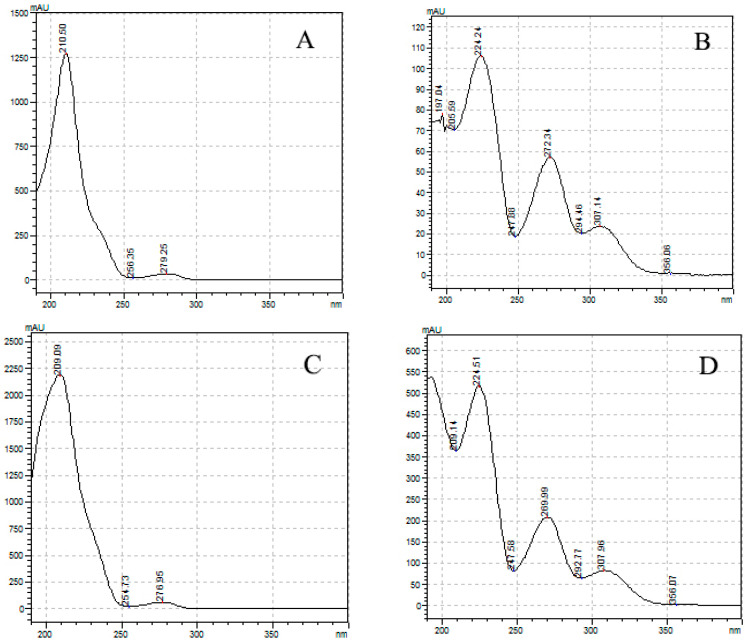
UV (ultraviolet) spectrum of four main cannabinoids. (**A**) Δ9-tetrahydrocannabinol, (**B**) Δ9-tetrahydrocannabinolic acid, (**C**) cannabidiol, and (**D**) cannabidiolic acid. Analysis was carried out from 190 nm, the lowest detection wavelength of the instrument.

**Figure 3:**
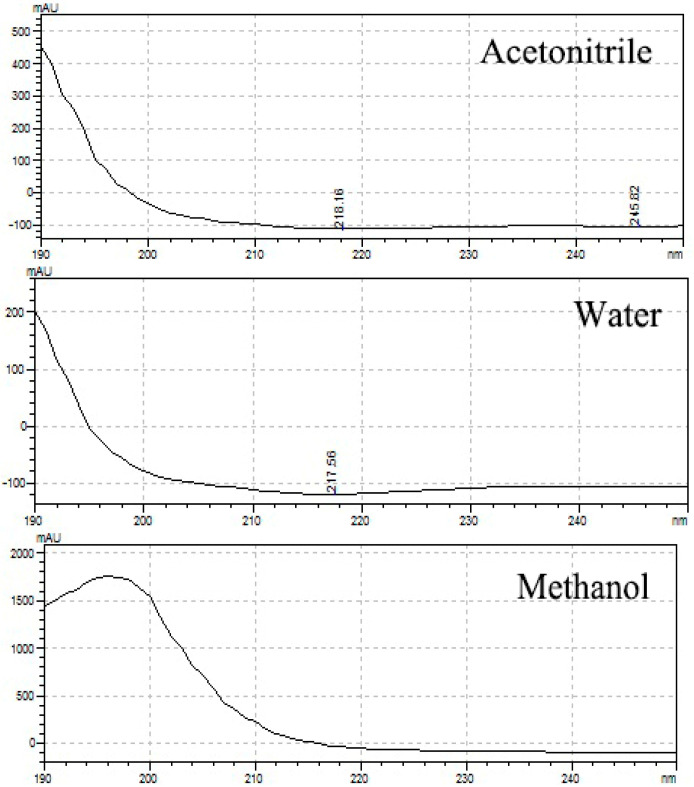
Absorbance spectrum of HPLC mobile phase; acetonitrile, methanol, and HPLC water. Analysis was carried out from 190 nm, the lowest detection wavelength of the instrument.

The absorbance spectrum of the standard solution in the UV region was measured and compared with the mAU value. As a result, Δ9-THC, Δ9-THCA, CBD, and CBDA had their maximum absorbance at 210.50, 221.24, 209.09, and 221.51 nm, respectively (Figure 2). Cannabinoids in the form of acidic precursors had relatively low absorbance in the UV region. The cut-off section of the mobile phase allowed us to judge whether it was easy to analyze at the selected wavelength (Figure 3). Acetonitrile and HPLC water showed an absorbance lower than or similar to that of the baseline at 220 nm, and methanol had a slightly higher absorbance than that of the baseline. This observation was consistent with that reported by Welch et al. [23]. We chose 220 nm as the optimal wavelength and acetonitrile as the organic mobile phase to facilitate sensitive peak detection, solvent absorption, and simultaneous analysis.

Peak tailing tends to lower the peak height and increase the limit of detection (LOD) and limit of quantitation (LOQ). As the area of the peak cannot be accurately determined, the reliability of quantification is automatically reduced. In the case of Δ9-THCA, which was eluted at the end, tailing occurred due to diffusion in the column and unstable kurtosis; therefore, the flow rate was set to 1.5 mL/min.

We used water as the polar solvent and 60%, 70%, 80%, and 90% as ACN. Separation was performed for 40 min using the isocratic method. Based on the last eluted THCA, 60% of cases were not detected within 40 min; at 70% RT, 10.314 (Δ9-THCA); 80% RT, 5.922 (Δ9-THCA); and 90% RT, 3.059 (Δ9-THCA). The peaks of all spiked standards except for those with 90% ACN were isolated; however, CBDA peak splitting occurred in the presence of 80% or higher ACN. Thus, 70% ACN was selected as the optimal solvent.

**Figure 4:**
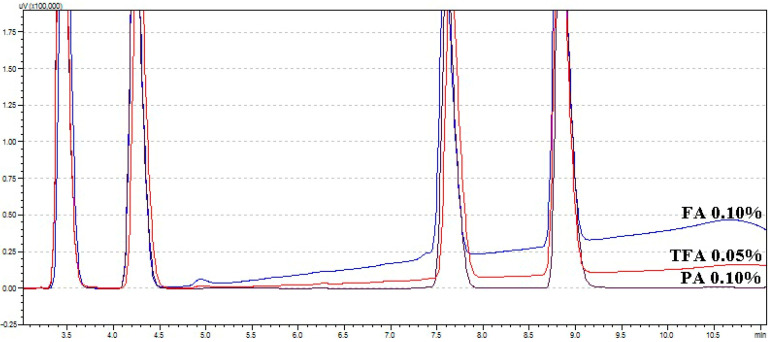
Analysis of four standards (100 μg/mL) under gradient conditions including acid. Blue line, 0.10% formic acid (FA); red line, 0.05% trifluoroacetic acid (TFA); black line, 0.10% phosphoric acid (PA).

The organic acids 0.05% TFA, 0.10% PA, and 0.10% FA were used under gradient conditions, and the gradient eluent set to simulation was used as the elution method to increase the elution rate (initiation, 70% ACN; 2 min, 70% ACN; 8 min, 85% ACN; 9 min, 70% ACN; 12 min, 70% ACN). FA and TFA showed a raised baseline (Figure 4). However, TFA was selected as the optimal organic acid because PA is not very compatible with mass spectrometry (MS), such as liquid-chromatography–mass-spectrometry (LC-MS) or liquid chromatography quadrupole time-of-flight (LC-Q-ToF).

The chromatography conditions were as follows: mobile phase A was water containing 0.05% TFA and mobile phase B was ACN containing 0.05% TFA. The gradient conditions were as follows: 70% B initially, 70% B for 2 min, 85% B for 8 min, 70% B for 9 min, and 70% B for 12 min. All four cannabinoids were measured at 220 nm using a UV–Vis detector. The flow rate was 1.5 mL/min. The injection volume was 10 μL, and the column oven temperature was set to 35 °C to prevent the destruction of acid-type cannabinoids and to reduce the retention time.

### 3.2. Validation

#### 3.2.1. System Suitability Test

A standard stock solution was prepared, and four types of standard products were mixed and measured nine times at the same concentration. The retention time did not exceed 2.00% of the relative standard deviation in % (RSD%) values for all four cannabinoids, and the peak area was less than 1.00%. The tailing factor was less than 2.0, and all theoretical plates had values of 1000 or more, thus meeting all USP standards. For resolution, CBDA, CBD, Δ9-THC, and Δ9-THCA with similar retention times were compared, thus showing excellent resolution (Table 1).

#### 3.2.2. Linearity

**Figure 5:**
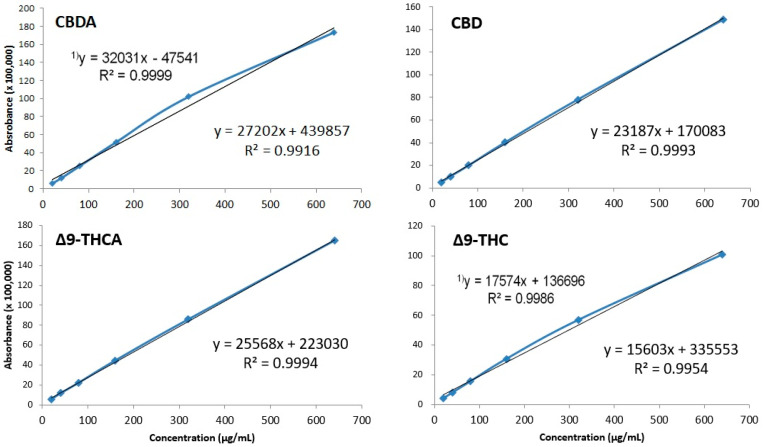
Calibration curves of four cannabinoids shown through the method of least squares. Regression equation and correlation coefficient with 640 μg/mL deleted. CBDA and Δ9-THC at 640 μg/mL were excluded from the quantification due to the violation of the Beer–Lambert law.

To test linearity, standard products were prepared at concentrations of 20, 40, 80, 160, 320, and 640 μg/mL, and calibration curves were measured using newly developed analysis methods. The coefficient of determination (R^2^) of the evaluated calibration curve was as follows: CBDA, R^2^ = 0.9916; CBD, R^2^ = 0.9993; Δ9-THC, R^2^ = 0.9944; and Δ9-THCA, R^2^ = 0.9994. Thus, we observed excellent linearity above R^2^ ≥ 0.9900. However, in the case of CBDA and Δ9-THC, the R^2^ values were 0.9999 and 0.9986, respectively, when the maximum concentration of 640 μg/mL was removed. This observation was associated with the high concentration, which violates the Beer–Lambert law because of the characteristics of the calibration curve. In the case of quantitative analysis, CBDA and Δ9-THC should be analyzed by excluding 640 μg/mL (Figure 5).

#### 3.2.3. Precision

To establish the precision of the analysis method, intra-day and inter-day analyses were performed over 2 days with five repetitions. The intra-day RSD% was 0.053–0.157 for CBDA, 0.641–1.203 for CBD, 0.689–1.329 for Δ9-THC, and 0.424–0.486 for Δ9-THCA. The inter-day RSD% for CBDA, CBD, Δ9-THC, and Δ9-THCA was 0.181, 1.944, 1.018, and 0.590, respectively.

#### 3.2.4. LOD and LOQ 

The LOD and LOQ were measured using the standard products at low concentrations of 10.0, 1.0, 0.5, and 0.1 μg/mL. The qualitative limit was judged to be effective when the signal/noise (S/N) value was at least 3.000, and it was <0.1 μg/mL for CBDA (S/N: 10.181), <0.5 μg/mL for CBD (S/N: 6.985), <0.5 μg/mL for Δ9-THC (S/N: 16.654), and <0.5 μg/mL for Δ9-THCA (S/N: 28.372). The LOQ was judged to be effective when the S/N value was at least 10.000, and it was found to be <0.1 μg/mL for CBDA (S/N: 10.181), <1.0 μg/mL for CBD (S/N: 13.272), <0.5 μg/mL for Δ9-THC (S/N: 16.654), and <0.5 μg/mL for Δ9-THCA (S/N: 28.372). All cannabinoids were detected at or below 0.5 μg/mL, and quantification was possible at or below 1.0 μg/mL.

### 3.3. Evaluation of Industrial Decarboxylation Efficiency of Hemp Inflorescence

**Figure 6:**
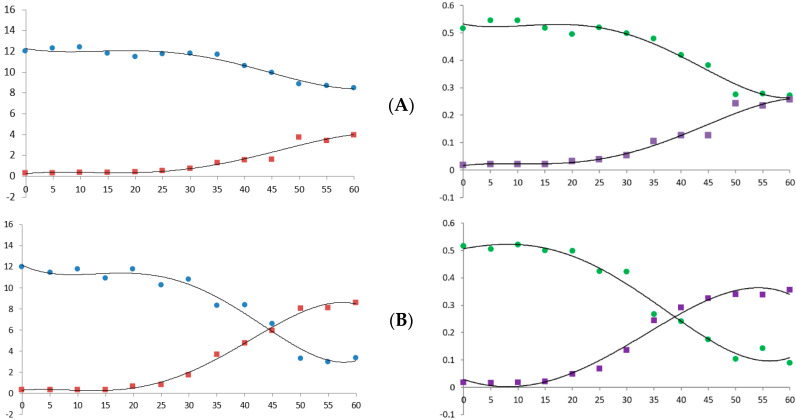

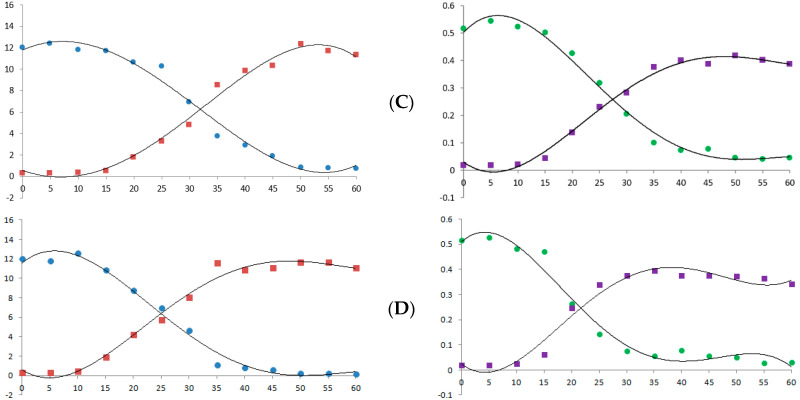
Decarboxylation curve of hemp flower powder with changes in temperature and time. (**A**) 90 °C; (**B**) 105 °C; (**C**) 120 °C; (**D**) 135 °C. X-axis, time (min); Y-axis, concentration (μg/mL, ×10,000). Blue dots, CBDA; red dots, CBD; green dots, Δ9-THCA; purple dots, Δ9-THC.

Heat is a major factor in cannabis decarboxylation [24]. Therefore, we analyzed the decarboxylation graph according to temperature and heating time using the newly established simultaneous analysis conditions. Figure 6 shows the decrease in the acidic form of cannabinoid and the increase in its neutral form. This decarboxylation reaction was accelerated as the temperature and time increased and showed a pattern similar to that reported in previous studies [21,25].

**Figure 7:**
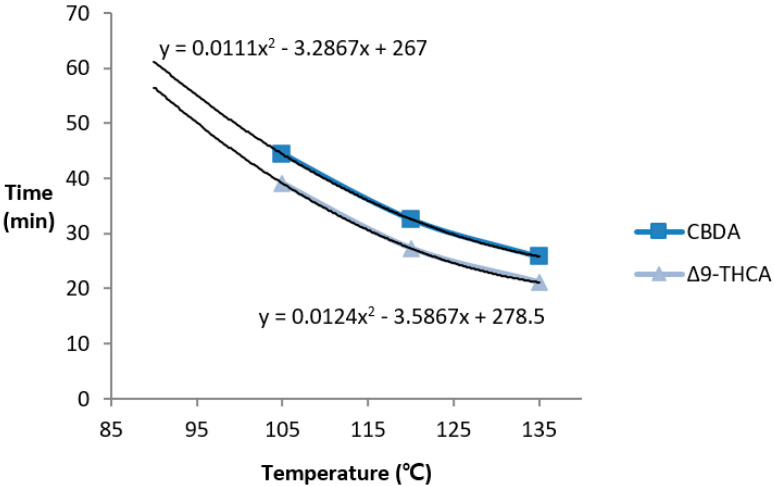
Time for 50% decarboxylation (DT_50_; when acidic cannabinoids are reduced to half) depending on the temperature. Δ9-THCA had a smaller DT_50_ value than CBDA did; decarboxylation occurred faster for THCA.

Hemp without additional processing also undergoes decarboxylation in its natural state owing to the effects of environmental factors such as light, and also generates neutral cannabinoids [26]. We selected the DT_50_ parameter (when acid cannabinoids are reduced by half; half-life) to accurately evaluate the rate of decarboxylation by removing the cannabinoid factors that are oxidized during heating and naturally generated neutral cannabinoids. DT_50_ was not achieved at 90 °C in either CBDA or Δ9-THCA after 60 min. The DT_50_ was 44.4 for CBDA and 39.1 for Δ9-THCA at 105 °C, 32.6 for CBDA and 27.3 for Δ9-THCA at 120 °C, and 25.8 for CBDA and 21.1 for Δ9-THCA at 135 °C. As for decarboxylation, Δ9-THCA had a DT_50_ value of approximately 5.1 ± 0.3 at the same temperature as CBDA, suggesting that Δ9-THCA decarboxylated faster than CBDA did under the same conditions (Figure 7).

The purpose of this study was to compare the loss of neutral cannabinoids from natural products and extracts in response to heating at a maximum temperature of 135 °C for 30 min using randomly selected CW21-13 and RDA-35 varieties. The DT_50_ values of the acidic cannabinoids were not noticeably different for both extracts and natural products. Thus, neither the extract nor the natural product had any effect on the decarboxylation rate. 

**Figure 8:**
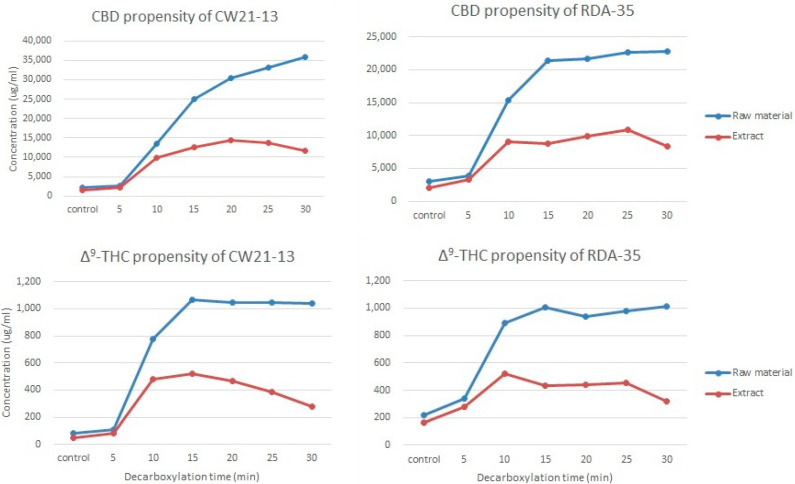
Loss of THC and CBD in different varieties at 135 °C for 30 min. Blue line, yield of raw material; red line, yield of extract. Samples were obtained every 5 min and analyzed, and all experiments were carried out simultaneously under the same conditions.

Upon incubation for 30 min at 135 °C, the yield of neutral cannabinoids increased in CW21-13. The CBD content of the raw material and extract was 33,786 and 10,046 μg/mL (about 3.4 times), respectively, and the Δ9-THC content was 956 and 235 μg/mL (about 4.0 times), respectively. In the case of RDA-35, the CBD and Δ9-THC contents of the raw material were 19,756 and 792 μg/mL, respectively, and those of the extract were 6306 μg/mL (about 3.1 times) and 155 μg/mL (about 5.1 times), respectively. Thermal destruction of neutral cannabinoids in the raw material was significantly lower in both varieties (Figure 8). Even during visual evaluation, the neutral cannabinoid levels increased over time in the extract and decreased in the second half. On the other hand, the natural product had a pattern of increase or did not continue to decrease even at 30 min, the end-point of the experiment.

## 4. Conclusions

We have successfully developed a fast and reliable analytical method using the widely employed HPLC–UV/Vis system. The validation tests demonstrated the high suitability of the method. The decarboxylation process of hemp was analyzed using this chromatographic method, which successfully isolated four major cannabinoids within 10 min. The overall decarboxylation rate may vary depending on factors such as the volume of the reactor due to the nature of the closed oven [27]. However, Δ9-THCA occurred more rapidly at a lower temperature than CBD did in natural products under the same conditions. The thermal destruction of cannabinoids occurred more in extracts than in natural products. The results of this study are of great value. This method allows for quick quantification and selection of the main cannabinoids in hemp breeding for industrial applications. In addition, it provides justification about the various processes that occur during the decarboxylation of hemp in its natural state.

## Figures and Tables

**Table 1:** Results of system suitability test (*n* = 9).

	Cannabinoid	1	2	3	4	5	6	7	8	9	Aver.	RSD%
Resolution	Δ9-THCA	5.995	5.971	5.99	5.98	5.997	5.984	5.987	5.987	5.983	5.986	-
	Δ9-THC											
	CBD	4.042	4.043	4.029	4.028	4.024	4.04	4.041	4.061	4.034	4.038	
	CBDA											
T. plate number	Δ9-THCA	25,869	25,501	25,438	25,804	25,857	25,816	25,420	25,769	25,804	25,698	-
	Δ9-THC	18,632	18,672	18,611	18,596	18,642	18,606	18,601	18,571	18,591	18,614	
	CBD	6914	6947	6806	6921	6838	6917	6911	6908	6911	6897	
	CBDA	5980	5893	5987	5987	5900	5983	5980	5973	5980	5963	
Tailing factor	Δ9-THCA	1.4	1.4	1.39	1.4	1.38	1.39	1.39	1.39	1.39	1.39	-
	Δ9-THC	1.38	1.38	1.38	1.38	1.37	1.37	1.37	1.37	1.38	1.38	
	CBD	1.34	1.34	1.34	1.35	1.35	1.35	1.34	1.32	1.35	1.34	
	CBDA	1.4	1.4	1.41	1.4	1.41	1.39	1.39	1.39	1.4	1.4	
Peak area	Δ9-THCA	1,500,678	1,497,089	1,494,576	1,489,975	1,498,975	1,509,757	1,509,785	1,498,546	1,509,901	1,501,031	0.484
	Δ9-THC	1,245,445	1,253,876	1,247,264	1,247,879	1,249,487	1,254,579	1,250,047	1,251,057	1,226,789	1,247,380	0.663
	CBD	477,667	477,348	473,487	476,402	477,259	472,008	476,834	472,695	479,546	475,916	0.539
	CBDA	1,775,767	1,779,756	1,766,487	1,769,487	1,767,534	1,776,789	1,779,785	1,773,246	1,775,875	1,773,858	0.282
Retention time	Δ9-THCA	8.815	8.82	8.809	8.804	8.813	8.806	8.806	8.798	8.804	8.808	0.076
	Δ9-THC	7.423	7.431	7.419	7.416	7.425	7.418	7.417	7.411	7.415	7.419	0.081
	CBD	4.204	4.214	4.206	4.206	4.216	4.205	4.203	4.202	4.203	4.207	0.119
	CBDA	3.384	3.392	3.386	3.386	3.394	3.385	3.384	3.382	3.384	3.386	0.118

## Data Availability

Not applicable.

## Sample Availability

Samples of the compounds are not available from the authors.
